# Strategies of seedlings to overcome their sessile nature: auxin in mobility control

**DOI:** 10.3389/fpls.2015.00218

**Published:** 2015-04-14

**Authors:** Petra Žádníková, Dajo Smet, Qiang Zhu, Dominique Van Der Straeten, Eva Benková

**Affiliations:** ^1^Department of Plant Systems Biology, Flanders Institute for Biotechnology, GhentBelgium; ^2^Department of Plant Biotechnology and Bioinformatics, Ghent University, GhentBelgium; ^3^Department of Physiology, Laboratory of Functional Plant Biology, Ghent University, GhentBelgium; ^4^Institute of Science and Technology Austria, KlosterneuburgAustria

**Keywords:** differential growth, auxin, apical hook, phototropism, gravitropism, hormonal crosstalk

## Abstract

Plants are sessile organisms that are permanently restricted to their site of germination. To compensate for their lack of mobility, plants evolved unique mechanisms enabling them to rapidly react to ever changing environmental conditions and flexibly adapt their postembryonic developmental program. A prominent demonstration of this developmental plasticity is their ability to bend organs in order to reach the position most optimal for growth and utilization of light, nutrients, and other resources. Shortly after germination, dicotyledonous seedlings form a bended structure, the so-called apical hook, to protect the delicate shoot meristem and cotyledons from damage when penetrating through the soil. Upon perception of a light stimulus, the apical hook rapidly opens and the photomorphogenic developmental program is activated. After germination, plant organs are able to align their growth with the light source and adopt the most favorable orientation through bending, in a process named phototropism. On the other hand, when roots and shoots are diverted from their upright orientation, they immediately detect a change in the gravity vector and bend to maintain a vertical growth direction. Noteworthy, despite the diversity of external stimuli perceived by different plant organs, all plant tropic movements share a common mechanistic basis: differential cell growth. In our review, we will discuss the molecular principles underlying various tropic responses with the focus on mechanisms mediating the perception of external signals, transduction cascades and downstream responses that regulate differential cell growth and consequently, organ bending. In particular, we highlight common and specific features of regulatory pathways in control of the bending of organs and a role for the plant hormone auxin as a key regulatory component.

## Introduction

To compensate for their sessile lifestyle, plants developed unique mechanisms that provide them with an unusual level of developmental plasticity. The adaptive behavior of plants is noticeable throughout their life cycle. Already during early skotomorphogenesis (development in darkness), seedlings of dicotyledonous plants form a hook-like structure at the apical part of the hypocotyl to shield their tender shoot apical meristem and cotyledons from damage while penetrating the soil (Raz and Ecker, 1999). This apical hook is maintained in a closed stage while seedlings grow through the soil, and rapidly opens when exposed to light. Also during photomorphogenesis (development in light), shoot organs maintain the ability to coordinate their growth. Plants manage to direct growth in response to the perceived light; a process studied and described by Darwin and Darwin (1881) as phototropism. Equally important to light-directed growth is the ability of plants to sense and adjust growth in response to gravity. Gravitropism is of the utmost importance for plant growth and survival, in particular after underground germination, when gravity is the sole cue to direct both root and shoot growth. In higher plants, shoots are negatively gravitropic (growing opposite to the direction of the gravity vector), whereas roots are positively gravitropic (growing in the direction of the gravity vector; Knight, 1806). Changes in the direction of the gravity vector are continuously sensed and both the root and shoot are able to rapidly bend and realign their growth to maintain a vertical growth direction. Interestingly, despite the diversity of cues and nature of responding organs, all different types of organ bending, whether triggered by gravity or light, share the same mechanistic principle based on differential cell growth.

The first hints on the molecular basis of the differential growth underlying organ bending date to Darwin’s early studies on coleoptiles. Based on the bending of coleoptiles toward unilateral light, Darwin and Darwin (1881) predicted the existence of a messenger molecule which is transported from the site of light perception at the tip of coleoptile toward the site of response where bending occurs. Later, it was demonstrated that an asymmetrical accumulation of the plant hormone auxin at the non-versus light-stimulated side correlates with differential cell growth and organ bending (Boysen-Jensen, 1911) and a model implementing a role for auxin and its asymmetric distribution in the regulation of plant tropic responses was proposed (Cholodny, 1927, 1928; Went, 1928). Although the molecular basis underlying tropisms was unknown at that time, the Cholodny–Went model was an important conceptual step in the general understanding of the principles of plant movements. The model delineated the main processes mediating plant bending in response to environmental stimuli which encompass local signal sensing, and inter- and intracellular signal transition toward the site of response. Hence, the identification of the molecular components and mechanisms involved in the coordination of differential growth, underlying organ bending in response to environmental stimuli, became one of the main milestones of following research.

In our review we will discuss the current knowledge on the pathways engaged in the control of tropic responses and apical hook development, the developmental process which is similar to tropic responses largely based on coordinated differential cell growth. Similarities and specificities of mechanisms mediating organ bending in response to environmental stimuli will be highlighted.

## Phototropism

Conversion of light into chemical energy during photosynthesis is essential for sustaining life on earth. Plants belong to the few species capable of such an energy transformation and by storing energy in carbohydrate molecules such as sugars, nutritional resources are generated for other organisms. To adjust their light absorption capacities, plants have evolved different light perception and signaling systems. Among them, phototropism or bending of an organ toward unidirectional blue light, is one of the most important adaptive responses. For the summary of the common and different factors involved in the regulation of phototropism see the **Table 1**.

**Table 1:** Mechanism of differential growth in phototropism, gravitropism, and apical hook development.

	Phototropism		Gravitropism		Apical hook	
	Shoot	Root	Shoot	Root	Formation	Opening
Signal triggering differential growth	- Blue light	- Blue light	- Gravistimuli	- Gravistimuli	- Unknown, gravistimuli (?)	- UV, blue, red, far-red light
Perception site	- Hypocotylapex, elongation zone	- Columella	- Endodermis	- Columella	- Unknown	- Unknown
Responding site	- Hypocotyl elongation zone	- Root elongation zone	- Hypocotyl elongation zone	- Root elongation zone	- Upper hypocotyl	- Upper hypocotyl
Signal perception (receptors)	- Photoreceptors (PHOT1, PHOT2, CRY1, CRY2)	- Photoreceptor (PHOT1)	- Amyloplast sedimentation	- Amyloplast sedimentation	- Unknown	- Photoreceptors (PHYA, PHYB, CRY1)
Other factors contributing to signal perception and transduction	- NPH3 - PIF4, PIF5 - Katanin - Microtubules	- NPH3	- Amyloplast (EAL1) - Starch (PGM, SEX1) - Endodermis (SGR1,7) - Vacuoles (SGR2,SGR3,ZIG/SGR4, SGR8/GRV2/KAM2, SGR9) - Actin (SGR9)	- Starch (PGM)	- HLS1	- Unknown
Main components of polar auxin transport coordinatingasymmetric auxin distribution	- Eﬄux carriers (ABCB19, PIN3)	- Eﬄux carriers (PIN1, PIN3, PIN2)	- Eﬄux carriers (PIN3)	- Influx carriers (AUX1) - Eﬄux carriers (PIN3, PIN2, PIN7)	- Influx carriers (AUX1/LAX3) - Eﬄux carriers (PIN3, PIN4, PIN7)	- Eﬄux carriers (ABCB1, ABCB19)
Factors involved in modulation of polarity of auxin transport after stimuli	- Signaling factors (PID, D6PK,PKS1, PKS2, PKS4, calmodulin (CAM4,5,7) - Biochemical signals (Ca^2+^)	- Signaling factors (PID,PP2A, PKS1) - Biochemical signals (Ca^2+^, InsP3)	- Signaling factors (ARF-GEF GNOM,PID, D6PK, PKS4) - Biochemical signals (Ca^2+^, InsP3, InsP6?) - Cellular structures (vacuoles, actin)	- Signaling factors (ARF-GEF GNOM, PID, PKS1) - Biochemical signals (Ca^2+^?, pH, InsP3,InsP6?) - Cellular structures (ER, actin (ARG1, ARL2))	- Signaling factors (ECHIDNA, WAG2)	- Unknown
Pattern of asymmetric auxin distribution	- Maximum at non-illuminated side	- Maximum at illuminated site (?)	- Maximum at lower gravi-stimulated side	- Maximum at lower gravi-stimulated side	- Maximum at concave side of hook	- Loss of auxin maximum
Components of auxin signalling mediating tropic response	- Auxin receptors (TIR1/AFB, ABP1) - Auxin response factors (NPH4/ARF7) - Aux/IAA repressors (IAA19, IAA29)	- Unknown	- Auxin response factors (NPH4/ARF7, ARF19) - Aux/IAA repressors (IAA14)	- Auxin receptors (TIR1/AFB, ABP1) - Auxin response factors (NPH4/ARF7, ARF19) - Aux/IAA repressors (IAA14, IAA19)	- Auxin receptors (TIR1/AFB) - Auxin response factors (NPH4/ARF7, ARF19) - Aux/IAA repressors (IAA3, IAA19) - Auxin responsive genes (SAUR19 -| PP2C-D) inhibits)	
Downstream targets and events	- Enhanced elongation of cells accumulating auxin and bending toward light	- Enhanced elongation of cells accumulated auxin and bending away from light	- Enhanced elongation of cells accumulating auxin and bending against gravistimulus	- Inhibited elongation of cells accumulating auxin and bending toward gravistimulus	- Inhibited elongation of cells accumulating auxin and apical hook formation	- Release of cell elongation (?)

*This table describes the common and different factors in the regulation of the three forms of differential growth, from trigger sensing to bending of the concerned organs, focussing on auxin transport and signaling as the basis of differential growth.*

### Shoot Phototropism

#### Light Sensing

Although already in Darwin and Darwin (1881) the tip of coleoptiles was predicted to be the site of light perception, the identity of the light receptor(s) mediating the phototropic bending remained unknown for more than 100 years.

The isolation of *Arabidopsis* mutants defective in the response to unilateral light such as *non-phototropic1* (*nph1*), *nph2*, *nph3* and *nph4* (Khurana and Poff, 1989; Liscum and Briggs, 1996) enabled the discovery of the receptors in the phototropic pathway. The *NPH1* gene was found to encode a flavoprotein kinase and confirmed to act as a UV-A/blue receptor (Huala et al., 1997; Christie et al., 1998). Later, its homolog *NPH1-LIKE1* (*NPL1*) was shown to contribute to the perception of blue light mainly at high fluence rates (Sakai et al., 2001). NPH1 and NPL1, later renamed to PHOTOTROPIN1 (PHOT1) and PHOTOTROPIN2 (PHOT2), are considered to be the major blue light receptors controlling phototropic responses (Briggs et al., 2001). Residual phototropic responses of *phot1phot2* mutants to blue illumination suggested the involvement of other photoreceptors. The latter proved to be CRYPTOCHROME1 (CRY1) and CRYPTOCHROME2 (CRY2), because the mutation of *CRY1* and *CRY2* in the *phot1phot2* mutant completely abolished the phototropic response (Ohgishi et al., 2004). The PHOT1 and PHOT2 function might also be partially replaced by phytochromes, the red/far-red sensitive light receptors, due to their ability to partially perceive blue light (Kami et al., 2010). Furthermore, activated phytochromes were shown to enhance the retention of PHOT1 and PHOT2 at the plasma membrane (Han et al., 2008), as well as to modulate the degree of phosphorylation of CRY1 and CRY2 (Ahmad et al., 1998), which indicates their indirect contribution to the regulation of phototropism.

Expression and protein localization of PHOT1 in coleoptile tips perfectly complied with the predicted perception site for phototropism (Knieb et al., 2004; Matsuda et al., 2011). In contrast, in dicotyledonous plants such as *Arabidopsis*, the expression pattern of *PHOT1* and *PHOT2* is much more extended and therefore the precise location of the perception site is less definite. Recent works indicated that the light perception site in *Arabidopsis* is indeed less strictly defined when compared to coleoptiles and might depend on the developmental status of the plant. As shown by Christie et al. (2011), light perception above and at the hypocotyl apex controls phototropic bending of de-etiolated *Arabidopsis* seedlings. In contrast, upon removal of the cotyledons and hypocotyl apex of etiolated *Arabidopsis* seedlings, the phototropic response remains unaffected. *PHOT1* expression in the hypocotyl elongation zone is, however, essential for proper bending (Preuten et al., 2013). Furthermore, tissue-specific expression of *PHOT1* in either the epidermis, cortex or endodermis suffices to trigger a full phototropic response, indicating that local activation of *PHOT1* might lead to seedling-wide signaling, presumably mediated through a non-cell autonomous type of signal (Preuten et al., 2013).

#### Light Signal Transduction to Coordinate Shoot Differential Growth

Although phototropins PHOT1 and PHOT2, Ser/Thr kinases belonging to the AGC family (Bögre et al., 2003), have been recognized as principal light receptors mediating the phototropic response, the immediate downstream events triggered by light remain largely unknown. Mutant screens and the identification of photoreceptor-interacting factors provided important insights in the signal transduction cascade upon light sensing (Motchoulski and Liscum, 1999; Sullivan et al., 2009).

NON-PHOTOTROPIC HYPOCOTYL 3 (NPH3) was identified in early screens for mutants defective in phototropism, and despite its essential regulatory function, the mechanism of NPH3 action remained obscure for a long time (Liscum and Briggs, 1996). Recent results indicated that NPH3 acts as a substrate adapter in the CULLIN-based E3 ubiquitin ligase CRL3^NPH3^ complex (Roberts et al., 2011) and mediates mono- and multiubiquitination of PHOT1 under low-intensity blue light. This posttranslational modification of PHOT1 might promote clathrin-dependent internalization of the photoreceptor. In contrast, high-intensity blue light was found to trigger polyubiquitination which marks PHOT1 for proteasome-mediated degradation. The predicted function of NPH3 fits well with the previously demonstrated direct interaction between NPH3 and PHOT1 (Motchoulski and Liscum, 1999) and the observation that blue light irradiation might induce rapid internalization of PHOT1 (Kaiserli et al., 2009). In light of these results, NPH3 might primarily act in fine-tuning the photoreceptor activity through modulation of PHOT1 subcellular trafficking and desensitization by degradation.

Since Darwin’s experiments, evidence accumulated in support of a crucial role of an asymmetric auxin distribution, depending on functional polar auxin transport, for a proper phototropic response (Briggs, 1963; Epel et al., 1992; Friml et al., 2002). Yet, what do we know about the events coordinating the asymmetric auxin distribution and phototropic response downstream of PHOT1-mediated light perception? Among molecular components controlling polar auxin transport in plants (for review, see Petrášek and Friml, 2009), two auxin eﬄux carriers, ATP-BINDING CASSETTE B19 (ABCB19; Christie et al., 2011) and PIN-like 3 (PIN3; Friml et al., 2002), emerged to be involved in the control of the phototropic response. Loss of ABCB19 function results in an enhanced phototropic response and its expression pattern in the upper part of the hypocotyl of de-etiolated seedlings largely overlaps with that of PHOT1. Efforts to characterize a functional link between the photoreceptor and ABCB19 revealed that PHOT1 transiently interacts with ABCB19. Through this direct physical interaction PHOT1 promotes ABCB19 phosphorylation, which consequently leads to the attenuation of its auxin transport activity. Based on these observations it has been proposed that PHOT1 restrains ABCB19 activity, thereby increasing the accumulation of auxin in and above the hypocotyl apex to prime lateral auxin fluxes that are channeled to the elongation zone by the auxin transporter PIN3, hereby coordinating differential cell growth (Christie et al., 2011). The proposed model nicely complies with the previously presented phosphorylation gradient model which was based on the finding that unilateral irradiation induces a gradient of PHOT1 autophosphorylation across oat coleoptiles, with the highest level of phosphorylation occurring on the irradiated side (Salomon et al., 1997).

In contrast, lack of PIN3 activity causes reduced sensitivity to unilateral light, and localization of this transporter at lateral membranes of hypocotyl endodermal cells hinted at PIN3 involvement in the lateral redistribution of auxin in the lower elongation zone of the hypocotyl (Friml et al., 2002). However, whether and how PIN3 might coordinate auxin redistribution in endodermal cells to form local maxima in the cortex and epidermal cells at the non-illuminated side of the hypocotyl remained an open question. Recent work (Ding et al., 2011) provided a plausible mechanistic model postulating a light-induced local modulation of PIN3-dependent transport. The authors demonstrated that unilateral light polarizes PIN3 in the endodermal cells, which severely attenuates the PIN3 signal at the outer- and maintains a strong signal at the inner lateral membrane of illuminated endodermal cells. In contrast, no polarization of PIN3 was found in non-illuminated cells. The PIN3 polarization occurs downstream of PHOT1-dependent light perception, however, unlike ABCB19, PIN3 was not confirmed as a substrate of the PHOT1 kinase, suggesting an indirect PHOT1-mediated regulation of PIN3 polarization. Interestingly, transcription of PINOID (PID) kinase, previously implicated in the control of PIN polarization through phosphorylation (Friml et al., 2002; Ding et al., 2011), was found to decrease in response to light. Based on these observations, a model was proposed in which a light-dependent control of PID activity accounts for differential phosphorylation and polar targeting of PIN3, which subsequently results in an asymmetric auxin distribution during the phototropic response. Besides PID, D6 PROTEIN KINASE (D6PK), another member of the AGC kinase family, was shown to play a role in the phototropic response. However, unlike PID, D6PK-mediated phosphorylation of PIN3 seems to have a main impact on its auxin transport activity. Hence, D6PK might, through control of overall PIN3 transport activity rather than PIN3 polarity, contribute to the regulation of the phototropic response (Willige et al., 2013; Zourelidou et al., 2014). Haga and Sakai (2012) dissected the phototropic responses in three categories, pulse-induced phototropism, time-dependent second positive phototropism, and continuous-light-induced second positive phototropism. Interestingly, PID – PIN3 mediated auxin gradients were found to control primarily the pulse-induced but not the continuous-light-induced phototropism thus suggesting the existence of two functionally distinct mechanisms; a PIN-dependent mechanism in which transient stimulation is sufficient to induce phototropism, and a PIN-independent mechanism that requires continuous light stimulation (Haga et al., 2014).

Other important components of the signaling cascade controlling the phototropic response are PHYTOCHROME KINASE SUBSTRATE1 (PKS1), PKS2, and PKS4. PKSs were identified in a screen for early blue light response genes (Lariguet et al., 2006) and genetic analyses confirmed their role in PHOT1- and PHOT2-dependent phototropism under weak intensities of blue light (Lariguet et al., 2006; Kami et al., 2014). PKSs localize to the plasma membrane and interact with PHOT1 and NPH3 (Lariguet et al., 2006). However, no indications for their regulation of the PHOT1 membrane localization and trafficking, resembling NPH3 function, could be found. Current analyses of auxin levels, rootward auxin transport and auxin signaling in *pks1*, *pks2,* and *pks4* suggested that PKSs might contribute to the regulation of the phototropic response through modulation of local auxin signaling or lateral auxin transport, alternatively (Kami et al., 2014).

An important part of the response to blue light includes the rapid modulation of ion homeostasis such as calcium (Ca^2+^; Baum et al., 1999; Harada and Shimazaki, 2007). An earlier study revealed that a transient rise in cytosolic Ca^2+^ is important for PHOT1-mediated inhibition of hypocotyl growth, and the increase in Ca^2+^ attributed to PHOT2 function is required for blue light induced phototropism (Folta et al., 2003; Zhao et al., 2013). Detailed characterization of the PHOT role in Ca^2+^ homeostasis revealed that whereas PHOT1 seems to primarily control Ca^2+^ transport through plasma membranes, PHOT2 is mainly involved in the activation of intracellular Ca^2+^ resources and transport of Ca^2+^ from vacuoles and the endoplasmic reticulum (ER; Zhao et al., 2013).

Apart from blue light, also auxin might affect cytosolic Ca^2+^ levels. Inhibition of auxin transport upon 1-*N*-naphthylphthalamic acid (NPA) treatment inhibited a cytosolic Ca^2+^ increase in response to blue light, while auxin treatment led to a dramatic increase in cytosolic Ca^2+^ (Zhao et al., 2013), which support the role of the functional polar auxin transport and auxin in the blue light induced increase of Ca^2+^. However, functional links between light-controlled Ca^2+^ levels and auxin in the regulation of the phototropic response are still largely hypothetical. Considering recent findings on the involvement of cytosolic Ca^2+^ in PIN polarity establishment (Zhang et al., 2011), light- and auxin-stimulated modulation of Ca^2+^ levels might bring about a feedback regulation of PIN3 polarity establishment during the phototropic response. On the other hand, observations that PKSs interact with several members of the calmodulin family such as CAM4, CAM5, and CAM7, hint at a role of Ca^2+^ as a trigger of unknown downstream targets in the signaling cascade underlying the phototropic response (Zhao et al., 2013).

#### Auxin Coordinates Phototropic Organ Bending

Translation of the light-induced auxin gradient to an appropriate molecular response requires a functional hormone signaling cascade. Defects in auxin perception caused by the lack of the auxin receptors TIR1/AFB as well as ABP1 activity compromise the phototropic response (Möller et al., 2010; Effendi et al., 2011). This indicates that both TIR1- and ABP1-dependent auxin transduction cascades contribute to the regulation of phototropism. Although not much is known about events downstream of ABP1, it is presumably involved in control of the subcellular distribution of PIN auxin transporters and proper auxin distribution during phototropic response (Robert et al., 2010; Xu et al., 2010). In addition, ABP1, through negative feedback on auxin responses as demonstrated by Tromas et al. (2013), might fine-tune auxin signaling-dependent responses. Several components of the TIR1-controlled auxin signaling network, comprising the Auxin Response Factor (ARF) transcriptional regulators and their Aux/IAA repressors, including NPH4/ARF7, MSG2/IAA19, and IAA29, were recognized for their regulatory role in phototropism (Liscum and Briggs, 1996; Tatematsu et al., 2004; Okushima et al., 2005; Sun et al., 2013).

Aux/IAAs appear to be an important convergence point with the phytochrome-dependent light-regulated pathway. Transcription of *IAA19* and *IAA29* was found to be under control of PHYTOCHROME INTERACTING FACTOR4 (PIF4) and PIF5 (Sun et al., 2013). Originally, PIFs were identified as phytochrome interactors, which are phosphorylated and rapidly degraded to activate photomorphogenesis after light exposure (Castillon et al., 2007; Jeong and Choi, 2013). Recently published results indicated that these phytochrome-interacting partners might at the same time act in the regulatory pathway that mediates the attenuation of auxin signaling at the illuminated side of hypocotyls (Sun et al., 2013).

Activation of the auxin signaling pathway induces the expression of molecular targets, which, downstream of auxin, take part in the coordination of differential cell growth and thereby hypocotyl bending. Surprisingly, very few auxin-regulated target genes involved in phototropism are known so far.

Essential elements for the adaptive response of plant growth to the environment are cytoskeletal rearrangements. The microtubule cytoskeleton is an important component of the complex framework controlling cell growth. Microtubule organization directs the deposition of cellulose to the cell wall, thus contributing to the coordination of cell expansion and its directionality (Zandomeni and Schopfer, 1994). In elongating hypocotyl cells, cortical microtubules are arranged in parallel arrays mostly perpendicular to the hypocotyl axis. In response to blue light, microtubules undergo a rapid rearrangement into an orientation parallel to the hypocotyl axis (Laskowski, 1990; Nick et al., 1990). Recent work of Lindeboom et al. (2013) showed that this light-stimulated reorientation of cortical arrays involves the phototropin-dependent activation of the katanin-mediated severing of microtubules. The defective phototropic response in mutants lacking katanin demonstrate that the local light-induced microtubule rearrangement is an important mechanism for a proper phototropic response, however, molecular and mechanistic links between the rapid cytoskeletal response to light and other, in particular auxin-related events need to be explored.

Although the role of actin in the growth of cells is less evident, there is evidence supporting its involvement therein (Baskin and Bivens, 1995; Baluska et al., 2003). Experiments in etiolated rice coleoptiles clearly demonstrated that actin microfilaments are responsive to irradiation as well as auxin treatment. However, the developmental relevance of this actin dynamics modulation is unknown so far (Holweg et al., 2004).

As differential cell growth constitutes the mechanistic basis for organ bending during phototropism, genes controlling plant cell growth might be among expected auxin targets. Growth of a plant cell is driven by internal turgor pressure, and is dependent on the ability of the cell wall to extend under pressure. Turgor pressure results from the hydrostatic pressure exerted on the cell wall by the cytoplasm and its increase above a threshold leads to cell expansion (Boyer et al., 1985; Cosgrove, 1993; Peters and Tomos, 1996; Schopfer, 2006). On the other hand, the cell wall is a complex composite material consisting of stiff cellulose microfibrils, which are tethered by xyloglucans and embedded in a matrix of hydrosoluble pectins and structural proteins (Cosgrove, 2005; Burton et al., 2010). Hence, genes involved in the control of cell growth either through modulation of hydrostatic pressure, or by modifications of cell wall properties, such as relaxation through wall loosening, could be candidates. Indeed, several target genes of NPH4/ARF7 action, whose expression levels increase on the elongating side of phototropically stimulated *Brassica oleracea* hypocotyls, were revealed by a transcriptomic approach (Esmon et al., 2006). EXPA1 and EXPA8, two members of the α–expansin family, which mediate cell wall extension (Esmon et al., 2006), might be relevant for the establishment of phototropic curvature through control of cell wall expansion.

### Negative Root Phototropism

While above-ground organs tend to bend toward blue light, underground organs such as roots typically turn away from the blue illumination. Interestingly, the same light receptor PHOT1 and downstream components such as NPH3, PKS, Ca^2+^, which control positive shoot phototropism as well as InsP3, are involved in negative root phototropism (Boccalandro et al., 2008; Salinas-Mondragon et al., 2010; Wan et al., 2012; Zhang et al., 2013). Similar to the shoot, unilateral blue light induces an asymmetric auxin distribution. However, while an auxin maximum forms at the shaded side of the shoot, auxin seems to accumulate in the meristem of epidermal cells at the illuminated side of the root (Zhang et al., 2013), although this conclusion in not fully supported by the work of Wan et al. (2012), indicating auxin accumulation at shaded side of root tip. Even though the pattern of auxin accumulation needs to be unambiguously resolved, theobserved asymmetry in auxin distribution hints at the involvement of a tightly controlled auxin transport in photo-stimulated roots. Indeed, auxin transport mutants such as *pin2* and *pin3*, display defects in the root phototropic response (Wan et al., 2012; Zhang et al., 2013, 2014). Moreover, studies on the regulatory pathway acting downstream of light to modulate auxin transport activity, revealed striking similarities with the mechanism controlling shoot phototropism. As in the shoot, unilateral blue-light triggers the polarization of PIN3 in a PHOT1-, PINOID-, and PHOSPHATASE2A- (PP2A) dependent manner. In the root columella cells, PIN3 localizes to the outer lateral membranes at the illuminated side of the root and this blue light-induced PIN3 polarization might promote auxin redistribution toward the illuminated side of roots (Zhang et al., 2013). These results indicate that similar to the shoot, blue light-induced PIN3 polarization contributes to the control of an asymmetric auxin distribution during the negative root phototropic response. Besides PIN3, the activity of other root auxin transport components is also light-regulated. As recently shown, PIN1 and PIN2, major facilitators of rootward and shootward auxin streams, respectively, change their subcellular localization at the root apical meristem in response to illumination (Wan et al., 2012; Zhang et al., 2014). Hence, through common perception pathways light can effectively coordinate the activities of major auxin transporters and therefore the auxin distribution to control phototropic responses in different organs.

## Gravitropism

As for their response to light, the correct recognition of the gravity vector by the root and shoot is of vital importance for plant growth and survival. In particular, during underground germination gravity is the key cue directing plant growth. Whereas root growth aligns with the gravity vector, the shoot is negatively gravitropic and grows against the gravity vector (Knight, 1806). For the summary of the common and different factors involved in the regulation of gravitropism see the **Table 1**.

### Gravity Sensing

Specialized cells for the perception of gravity are called statocytes. In plants, these cells hold starch-containing plastids, referred to as amyloplasts, which sediment in the direction of gravity. Already back in Haberlandt (1900) and Němec (1900), amyloplasts were proposed to function as statoliths in gravity perception. This starch-statolith hypothesis is now widely accepted as a model for gravity perception in both roots and shoots of higher plants. However, different cell types function as statocytes in roots and shoots.

The root cap is essential for gravity perception in roots. Root cap removal through surgical ablation or genetic manipulation prevented gravitropic curvature of the root (Younis, 1954; Juniper et al., 1966; Tsugeki and Fedoroff, 1999) and high-precision laser ablation indicated the importance of the inner two cell layers of the root cap columella (Blancaflor et al., 1998). The perception of gravity outside the root cap was suggested by a continued gravitropic curvature of the root, while retaining a specific segment of the root elongation zone at a gravistimulated angle, even after a vertical orientation of the root cap (Mullen et al., 2000; Wolverton et al., 2002). However, gravity perception outside the root cap probably contributes only in a minor extent to the root gravitropic response.

In the shoot, starch-containing amyloplasts are found in the endodermis (Fukaki et al., 1998). The function of endodermal cells as statocytes is supported by several genetic studies. The *Arabidopsis endodermal-amyloplastless* (*eal1*) mutant, which lacks endodermal amyloplasts, exhibits hampered shoot gravitropism (Fujihira et al., 2000). Similarly, mutants in *SCARECROW* (*SCR*)/*SHOOT GRAVITROPISM1* (*SGR1*) and *SHORT*-*ROOT* (*SHR*)/*SGR7,* transcription factors of the *GRAS* gene family, which are indispensable for normal shoot and root endodermis formation, are characterized by a deficient shoot gravitropic response. The deficient shoot gravitropism in the latter mutants supports the function of shoot endodermal cells as statocytes in gravity perception. On the other hand, the unaffected root gravitropism in endodermis defective mutants hints at a difference in the site of gravity perception between shoots and roots, supporting the role of columella cells as primary perception site in the root (Benfey et al., 1993; Fukaki et al., 1996, 1998; Yamauchi et al., 1997).

Upon gravistimulation, amyloplasts sediment to the gravity-associated bottom of the statocytes and trigger responses resulting in gravitropic bending (Sack et al., 1986; Saito et al., 2005; Leitz et al., 2009). The dense starch accumulation in amyloplasts suggests an important function of starch biosynthesis for gravitropism. In the *Arabidopsis phosphoglucomutase* (*pgm*) mutant, amyloplast sedimentation is dramatically affected due to a low starch content, which correlates with a reduced gravitropic response in roots as well as shoots (Caspar and Pickard, 1989; Kiss et al., 1989, 1997; Weise and Kiss, 1999). Restoration of the gravitropic response in the *pgm* mutant upon exposure to a hypergravity stimulus indicates that the amyloplast starch levels might be an important factor in the gravity sensing (Fitzelle and Kiss, 2001). This is further supported by the *starch excess 1* (*sex1*) mutant phenotype. Due to a defective starch breakdown, amyloplasts are larger in the endodermal cells of light-grown *sex1* shoots, while being unaffected in the root columella cells (Caspar et al., 1991). Consistently, gravitropic sensitivity increases dramatically in light-grown shoots, whilst remaining unaltered in the root (Vitha et al., 2007). Altogether, these results indicate that amyloplasts rather than the enclosed starch function as statoliths. However, starch promotes amyloplasts sedimentation and therefore represents an important factor in the control of the gravitropic response (Morita, 2010).

Amyloplast movements differ substantially between root and shoot statocytes. In roots, amyloplast sedimentation invariably occurs at the gravity-associated bottom of the statocytes (Yoder et al., 2001). In shoots, amyloplasts often exhibit dynamic saltatory movements rather than a static sedimentation, and are more unlikely to sediment to the gravity-associated statocytes’ bottom (Saito et al., 2005; Hashiguchi et al., 2013). Shoot statocytes contain large vacuoles and amyloplasts are enclosed by transvacuolar strands, i.e., cytoplasm enclosed by the tonoplast (Clifford et al., 1989). Amyloplast sedimentation through the vacuolar strands of shoot statocytes suggests vacuole involvement in gravity perception (Lawton et al., 1986; Clifford et al., 1989; Morita et al., 2002; Saito et al., 2005). Indeed, the phenotype of mutants in *SGR2*, *SGR3*, *ZIG*/*SGR4*, and *GRV2*/*SGR8*/*KAM2*, encoding proteins involved in membrane trafficking, vacuole formation and functioning, supports the importance of the central vacuole in shoot gravity sensing (Kato et al., 2002; Morita et al., 2002; Yano et al., 2003; Saito et al., 2005; Silady et al., 2008; Hashiguchi et al., 2013). The fact that vacuoles are Ca^2+^ and H^+^ reservoirs, might hint at an indirect role of vacuoles in gravity sensing and signal transduction (Perbal and Driss-Ecole, 2003). Root gravitropism remains unaffected in the *sgr2*, *sgr3*, *zig/sgr4*, and *grv2/sgr8/kam2* mutants, excluding the importance of vacuoles in gravity perception in the root (Kato et al., 2002; Morita et al., 2002; Yano et al., 2003; Silady et al., 2008). Root columella cells exhibit a large ER, small vacuoles and abundant cytoplasm, and amyloplasts are not enclosed by the vacuolar membrane and thus sediment independently from the vacuole (Saito et al., 2005). The ER could fulfil the role of Ca^2+^ and H^+^ reservoir and amyloplast-ER interactions could mediate the signal transduction after gravity perception in the root (Perbal and Driss-Ecole, 2003). The role of Ca^2+^ and H^+^ as signals involved in the control of gravitropism will be discussed in detail below.

Actin filaments have been identified as important mediators of gravitropism (reviewed by Blancaflor, 2002). They occur within the transvacuolar strands in shoot statocytes and surround amyloplasts in root statocytes (Traas et al., 1987; Tominaga et al., 2000; Saito et al., 2005). The enhanced root and shoot gravitropism upon disruption of actin microfilaments indicates an inhibitory function (Yamamoto and Kiss, 2002; Hou et al., 2004; Palmieri and Kiss, 2005; Saito et al., 2005). The negative role of actin in gravisensing is supported by the observation that saltatory movements of shoot endodermal amyloplasts are increased in the *Arabidopsis* mutant *sgr9* and that amyloplasts do not sediment but rather move around, or cluster together, entangled in actin filaments (Nakamura et al., 2011). Disruption of actin in *sgr9* by either Latrunculin B or mutation of *FRIZZYSHOOT1* (*FIZ1*), involved in actin microfilament formation (Kato et al., 2010), restores normal amyloplast sedimentation and thus normal shoot gravitropism. SGR9, a RING-type E3 ligase, presumably regulates the dissociation of amyloplasts from the actin microfilaments and thereby control the balance between amyloplast sedimentation and saltatory movements in the shoot endodermal cells (Nakamura et al., 2011). Hence, actin microfilaments probably regulate gravity perception by fine-tuning the dynamics of amyloplast sedimentation and saltatory movements.

### Signal Transduction

Amyloplast sedimentation in response to gravity is considered to excite a biochemical signal that converts gravity perception into gravitropic curvature. Rapid modulation of the cytoplasmic pH, Ca^2+^ and inositol 1,4,5-triphosphate (InsP3) levels in response to a gravistimulus qualified them as early signaling molecules. How these signaling molecules might coordinate gravitropic bending and possibly the asymmetric redistribution of auxin, the hormonal signal coordinating differential cell growth, will be discussed in the following paragraph.

#### Coordinated Auxin Redistribution During Gravitropic Responses

Similar to phototropism, gravitropic curvature results from a differential auxin distribution (Cholodny, 1927; Went, 1928; Evans, 1991). In gravistimulated roots, auxin accumulates in the epidermal cells at the lower side of the root apex and coordinates bending of the root through the inhibition of cell elongation (Friml et al., 2002; Ottenschläger et al., 2003). Monitoring of the auxin dynamics using the auxin sensitive reporter (DII)-VENUS demonstrated that auxin rapidly relocalizes to the lower side of roots within minutes after gravistimulation (Band et al., 2012; Brunoud et al., 2012). Research on the mechanism controlling this rapid auxin redistribution upon gravistimulation, provided a detailed picture on the directionality of the auxin flow, and demonstrated that the auxin transport machinery acting at the root tip was instrumental (Bennett et al., 1996; Gälweiler et al., 1998; Luschnig et al., 1998; Müller et al., 1998; Friml et al., 2002; Swarup et al., 2005; Harrison and Masson, 2008). Auxin is transported acropetally through the central vasculature toward the root tip. Auxin transporters including the AUX1 influx carrier and the PIN1, PIN4, PIN7 as well as ABCB1 and ABCB19 eﬄux carriers procure the auxin movement in this direction. In the columella, AUX1, PIN3 and PIN2 regulate the basipetal auxin transport through the lateral root cap and epidermis toward the root elongation zone where the auxin distribution controls the dynamics of cell growth. However, what are the mechanisms triggering the differential auxin transport resulting in local auxin maxima at the lower side of the root elongation zone upon gravistimulation? The agravitropic phenotype of the *aux1, pin2, pin3* and *pin3, pin7* mutants delineated *AUX1*, *PIN2*, *PIN3* and *PIN7* as key transporters coordinating the auxin redistribution upon gravistimulation. Subsequent studies on the polar membrane localization and subcellular dynamics of the auxin transporters, revealed that within a few minutes after gravistimulation, the PIN3 and PIN7localization changed from apolar to polar with maxima at the bottom of root columella cells. This rapid PINs relocation might increase the efficiency of the auxin flow toward the lower side of the gravistimulated root (Friml et al., 2002; Kleine-Vehn et al., 2010). Beyond the root columella, AUX1 and PIN2 procure auxin transport through the lateral root cap and epidermis toward the root elongation zone, where the accumulation of auxin limits cell expansion (Luschnig et al., 1998; Müller et al., 1998; Swarup et al., 2005).

Similar to the root, auxin redistribution upon shoot gravistimulation depends on the activity of the polar auxin transport machinery (Rakusová et al., 2011). In the *pin3* mutant, the asymmetrical auxin distribution in gravistimulated hypocotyls is significantly reduced and gravitropic responses are limited. Consistently, upon gravistimulation PIN3 relocalizes to the lower side of the endodermal cells where it mediates an asymmetrical auxin distribution, resulting in auxin accumulation to the lower side of the gravistimulated shoot (Friml et al., 2002; Rakusová et al., 2011).

Redirection of the auxin stream after gravistimulation requires a rapid adaptation of the auxin transport machinery. PIN3 and PIN7 polarization in both roots and PIN3 in shoots presumably occurs via ARF-GEF GNOM-dependent protein transcytosis (Geldner et al., 2003; Kleine-Vehn et al., 2010; Rakusová et al., 2011). Upon gravistimulation PIN3 polarization was demonstrated to rest also on PID kinase-dependent regulation. PID overexpression impeded gravity-induced PIN3 polarization and consequently caused a defective shoot gravitropism, whereas hypocotyl growth remained unaltered. On the other hand, in *pid* loss-of-function seedlings gravity still induced an asymmetrical PIN3 localization (Rakusová et al., 2011). Apart from PID, D6PK, also a member of the AGC kinase family, was shown to regulate shoot gravitropism. Shoot gravitropism is defective in *d6pk* mutants. The reduced phosphorylation of PIN3, suggest D6PK involvement in shoot gravitropism through regulation of PIN3 phosphorylation and therefore auxin transport (Zourelidou et al., 2009; Willige et al., 2013). D6PK levels at the plasma membrane correlate with PIN3 phosphorylation and consequently PIN3-dependent auxin eﬄux. Plasma membrane D6PK abundance depends on ARF-GEF GNOM activity (Barbosa et al., 2014), which is in line with the dependency of PIN3 polarization on ARF-GEF GNOM in shoot gravitropism (Geldner et al., 2003; Kleine-Vehn et al., 2010; Rakusová et al., 2011). D6PK polarity is independent from PID (Barbosa et al., 2014), excluding D6PK function directly down-stream from PID in the regulation of auxin transport in shoot gravitropism. Similarly, PIN2-mediated auxin transport is rapidly altered to promote auxin redistribution upon gravistimulation of the root. Whereas the PIN2 level increases in the epidermal cells at the lower side of the root apex, at the upper side PIN2 undergoes endocytosis and lytic degradation (Abas et al., 2006). Again, PID kinase was shown to regulate auxin transport and gravitropism by targeting the PIN2 auxin eﬄux carrier in the root. The *pid-9* mutant is characterized by reduced PIN2-mediated basipetal auxin transport and gravitropism. However, in contrast with PID overexpression, the asymmetrical auxin distribution of PIN2 upon gravistimulation remained unaltered in the *pid-9* mutant, suggesting a PIN2 polarity-independent regulation of auxin transport during gravitropism (Sukumar et al., 2009). Similar to negative phototropism in the root, PHYTOCHROME KINASE SUBSTRATE1 (PKS1) seems also involved in root gravitropism. However, whilst PKS1 positively regulates root phototropism, root gravitropism is counteracted by PKS1. The gravitropic response of etiolated roots is enhanced in the *pks1* mutant, whereas it is slightly reduced upon overexpression of *PKS1* (Boccalandro et al., 2008). On the other hand, shoot gravitropism is normal in etiolated *pks1pks2pks4* mutants (Lariguet et al., 2006). Overexpression of *PKS4*, however, inhibited shoot gravitropism whilst upon mutation of *PKS4* the gravitropic response of etiolated seedlings was not substantially affected (Schepens et al., 2008), suggesting redundancy among the PKS kinases. Hence, the PKS family plays important functions during light-regulated tropic growth responses, whereas in darkness their involvement in gravitropism seems minor and requires more research.

#### Biochemical Signals Involved in Signal Transduction Upon Gravity Sensing

Although full understanding of the molecular mechanisms controlling gravitropic bending and auxin transport modulation after a gravistimulus is still lacking, there are several indications for the involvement of signaling molecules such as Ca^2+^ and InsP3 (inositol1,4,5-trisphosphate) in these regulations.

Calcium has been implicated as a signal in the transduction of gravity sensing to differential growth (Sinclair and Trewavas, 1997; Fasano et al., 2002). Gravistimulation induces an increase in the cytoplasmic Ca^2+^ in the shoot within seconds (Legué et al., 1997; Plieth and Trewavas, 2002; Toyota et al., 2008, 2013). In roots, however, it remains obscure whether an increase in cytoplasmic Ca^2+^ occurs upon gravistimulation (Legué et al., 1997; Toyota et al., 2008). A recent study of Monshausen et al. (2011) reports asymmetrical transient Ca^2+^ changes, with Ca^2+^ levels decreasing and increasing at the upper and lower site of gravistimulated roots respectively. These changes in the Ca^2+^ concentration are, however, much slower compared to shoot gravitropism (minutes compared to seconds). Furthermore, the dependency of both root and shoot gravitropism on Ca^2+^ signaling is supported by the defective gravitropism upon administration of Ca^2+^chelators (Lee et al., 1983; Daye et al., 1984; Millet and Pickard, 1988; Toyota et al., 2008). Curvature of the root in response to unilateral administration of Ca^2+^ also suggests the involvement in differential growth (Hasenstein and Evans, 1988; Ishikawa and Evans, 1992).

Another signaling molecule implicated in gravity sensing and gravitropic curvature is InsP3 (Perera et al., 1999, 2001). Upon gravistimulation, InsP3 levels temporarily increase in *Arabidopsis* and oat inflorescence stems before returning to basal levels (Perera et al., 2001, 2006). Furthermore, the inhibition of InsP3 production substantially attenuates the graviresponse in *Arabidopsis* roots and oat shoots (Perera et al., 2001; Andreeva et al., 2010; Salinas-Mondragon et al., 2010). Consistently, inhibition of InsP3-mediated signaling postpones and reduces the gravitropic response of *Arabidopsis* roots and shoots (Perera et al., 2006; Smith et al., 2013). Nevertheless, no InsP3 receptor was yet identified in plants (Wheeler and Brownlee, 2008; Munnik and Testerink, 2009). InsP3 is an important intermediate in the biosynthesis of InsP6 (inositol hexakisphosphate; Testerink and Munnik, 2011). Furthermore, both InsP3 and InsP6 are able to induce the release of Ca^2+^ (Krinke et al., 2007; Im et al., 2010; Gillaspy, 2011). Hence, the question remains whether InsP6 rather than InsP3 functions as signaling molecule in gravitropism. The root of the *mips1* (*myo*-Inositol-1-phosphate synthase) mutant, which is characterized by reduced levels of InsP6 (Raboy, 2003), is agravitropic (Chen and Xiong, 2010). The absence of a significant difference in gravitropic root curvature between *pin2* and the *pin2mips1* double mutant, suggests that the defective root gravitropism in *mips1* is due to dysfunction of PIN2, which plays a key role in auxin redistribution upon gravistimulation. This is supported by the slower PIN2 aggregation in *mips1* upon Brefeldin A treatment, indicative of an altered PIN2 trafficking (Chen and Xiong, 2010). Further research is required to elucidate whether InsP6 rather than InsP3 or both, are more important in gravitropism.

The function of InsP3 in gravitropic signaling could be linked to that of Ca^2+^. Gravistimulation could induce IsnP3 production, which in turn could inducea Ca^2+^ release (Ghosh et al., 1988; Gilroy et al., 1990; Perera et al., 2006). This is supported by the corresponding changes in InsP3 and Ca^2+^ levels upon gravistimulation (Toyota et al., 2008, 2013). Hence, through Ca^2+^- and/or InsP3-induced Ca^2+^ release, a sustained Ca^2+^ increase could be elicited mediating gravitropic signaling (Toyota et al., 2013). The opening of stretch-activated Ca^2+^ channels by the mechanical strain exerted by sedimenting amyloplasts on actin microfilaments, could be a mechanism for gravistimulation-induced Ca^2+^ release (Perbal and Driss-Ecole, 2003). In root statocytes the large ER could function as an intracellular Ca^2+^ reservoir, which upon contact with sedimenting amyloplasts could release Ca^2+^. In shoot statocytes on the other hand, the large central vacuole could function as an intracellular Ca^2+^ reservoir, releasing Ca^2+^, possibly upon contact with the sedimenting amyloplasts or through actin filament-activated mechanosensitive Ca^2+^ channels (Perbal and Driss-Ecole, 2003).

Recent works indicated that Ca^2+^ and InsP3 might control gravitropic responses through the modulation of PIN polar targeting and auxin distribution (Lee and Evans, 1985; Plieth and Trewavas, 2002; Benjamins et al., 2003; Zhang et al., 2011). Firstly, mutation in *INOSITOL POLYPHOSPHATE 5-PHOSPHATASE 13* (*5PTase13*), encoding an enzyme involved in phosphatidylinositol metabolism, was found to enhance the root gravitropic response. The reduced sensitivity of the *5PTase13* mutant to the polar auxin transport inhibitor NPA, denoted the involvement of enhanced polar auxin transport and consistently, an altered PIN2 distribution, with an expression extended to the root elongation zone in the *5PTase13* mutant, has been observed (Wang et al., 2009). Another indication for the role of InsP3 and Ca^2+^ in the control of the PIN-dependent auxin distribution during gravisignaling emerged from a screen for suppressors of the PIN1 gain-of-function phenotype (Zhang et al., 2011). The ectopic presence of basally localized PIN1 in the epidermal cells resulted in root agravitropism, which was suppressed after mutation of *suppressor of PIN1 overexpression* (*SUPO1*). *SUPO1* (*SAL1/FRY1/HOS2/ALX8/RON1*) was shown to code for a protein with inositol polyphosphate 1-phosphatase and 3′(2′),5′-bisphosphate nucleotidase activities (Zhang et al., 2011). Elevated cytosolic InsP3 and Ca^2+^ levels, either due to the lack of SUPO1 activity or as result of pharmacological treatment, were found to modify the PIN polar membrane localization and consequently the root gravity response (Zhang et al., 2011). Altogether, these findings hint at the role of InsP3-mediated Ca^2+^ release in the control of PIN-mediated auxin distribution and subsequently auxin-dependent gravitropism.

Apart from an increase in both Ca^2+^ and InsP3, gravistimulation also induces a rapid increase in the cytoplasmic and cell wall pH of root cap columella cells (Scott and Allen, 1999; Fasano et al., 2001; Johannes et al., 2001). In the *Arabidopsis pgm* mutant, cytoplasmic alkalization upon gravistimulation is, however, inhibited, indicating the dependency on amyloplast sedimentation. Furthermore, the *Arabidopsis* mutant *arg1* is defective in the gravity-induced cytoplasmic pH changes within the root columella cells, partly explaining the altered root gravitropic response of the mutant (Boonsirichai et al., 2003). Although the increase in cytoplasmic pH in the root cap columella cells is probably important for root gravitropism, the spatial and temporal uniformity of the pH changes in the root cap columella cells indicate that pH changes do not transduce directional gravity information (Fasano et al., 2001).

The function of actin microfilaments in gravitropism is not confined to gravisensing (discussed above). Modulation of the polar membrane localization of auxin transporters after a gravistimulus is largely dependent on the actin cytoskeleton (Geldner et al., 2001). Stabilization of actin microfilaments limits the subcellular dynamics of auxin transporters and consequently inhibits the asymmetrical auxin accumulation and the root gravitropic response (Dhonukshe et al., 2008). The *Arabidopsis ALTERED RESPONSE TO GRAVITY 1* (*ARG1*) and *ARG1-LIKE 2* (*ARL2*) genes further strengthen the role of actin in the regulation of PIN dynamics. ARG1 interacts with the cytoskeleton, supposedly with actin microfilaments, and *arg1* and *arg1-like 2* mutants exhibit a reduced shoot and root gravitropism, as well as defects in the asymmetrical auxin redistribution (Fukaki et al., 1997; Sedbrook et al., 1999; Boonsirichai et al., 2003; Guan et al., 2003; Harrison and Masson, 2008). Furthermore, rearrangements of the actin cytoskeleton during cell expansion after gravistimulation also suggest its involvement as a regulator of cell elongation (Pozhvanov et al., 2013).

### Auxin Coordinates Gravitropic Bending

Similar to phototropism, the accumulation of auxin in gravistimulated organs triggers downstream responses through a transduction cascade consisting of the TIR1/AFB auxin receptor family, ARFs and the Aux/IAA repressor family. Several components of the auxin signaling pathway were demonstrated to contribute to the regulation of the gravitropic response in both shoot and root (reviewed by Masson et al., 2002). Auxin regulates its own eﬄux during root gravitropism by affecting the abundance of PIN proteins. PIN2 vacuolar targeting and degradation depends on a (SCFTIR1/AFB)-mediated signaling (Baster et al., 2013), whereas PIN2 endocytosis depends on ABP1-mediated signaling (Paciorek et al., 2005). The PIN2 turnover is the result from biosynthesis, endocytosis and vacuolar targeting and degradation. Upon gravistimulation, this PIN2 turnover is differentially regulated by auxin at the upper and lower side of the root to promote its eﬄux at the lower side, and to inhibit its eﬄux at the upper side of the gravistimulated root (Paciorek et al., 2005; Baster et al., 2013). Mutation of the *MASSUGU 2* (*MSG2*)/*IAA19* gene results in a reduced hypocotyl gravitropic response, whereas growth and developmental processes other than differential growth are unaffected (Watahiki and Yamamoto, 1997; Tatematsu et al., 2004). Likewise, mutation of *SOLITARY ROOT (SLR)/IAA14*, results in agravitropic roots and shoots (Fukaki et al., 2002). The severely abolished auxin-dependent up-regulation of PIN gene expression in the *slr1/iaa14* mutant (Vieten et al., 2005), suggests an Aux/IAA-dependent regulation of auxin transport upon gravitropism. Furthermore, an asymmetrical auxin redistribution in the root in response to gravistimulation triggers the expression of the auxin response factor ARF19 (Band et al., 2012). After mutation of *ARF7/NPH4* and *ARF19,* both hypocotyl and root are characterized by an agravitropic phenotype (Okushima et al., 2005), reminiscent of the phenotype of the *slr/iaa14* mutant (Fukaki et al., 2002). However, in contrast to the *arf19* single mutant, the *arf7/nph4* single mutant is characterized by a reduced shoot gravitropism. Furthermore, neither of these mutations conferred an agravitropic root phenotype (Liscum and Briggs, 1996; Ruegger et al., 1997; Harper et al., 2000; Okushima et al., 2005; Li et al., 2006). *ARF7*/*NPH4* and *ARF19* are expressed in both the root and the shoot. *ARF19* expression is stronger in the root, whereas *ARF7*/*NPH4* expression is stronger in the shoot (Okushima et al., 2005; Wilmoth et al., 2005; Li et al., 2006). The normal root gravitropic response of the single mutants indicates that these ARFs act largely redundant in the root. In contrast, the stronger expression of *ARF7*/*NPH4* in the shoot, is consistent with the stronger effect of *arf7/nph4* on shoot gravitropism, indicating that these ARFs function less redundantly compared to the root. The induced expression of common auxin responsive genes by ARF7/NPH4 and ARF19 supports their redundant functions in gravitropism (Okushima et al., 2005; Wilmoth et al., 2005). Furthermore, whereas the *nph4/arf 7* single mutant exhibited no obvious reduction, the auxin-dependent relocation of PIN1 and PIN2 was reduced in the *arf7 arf19* double mutant (Sauer et al., 2006), which supports an ARF-dependent auxin transport in root gravitropism. Altogether these results support an Aux/IAA-ARF-dependent regulation of gravitropism, presumably partly through the regulation of auxin transport. Despite evidence for the involvement of several components of the auxin signaling cascade in the control of gravitropic bending, clarification of downstream targets and mechanisms that regulate differential cell growth requires deeper investigation.

## Apical Hook Development

Another remarkable example of tightly coordinated differential growth in plants is the development of the apical hook. Typically, dicotyledonous plants form a hook-like structure, which protects the delicate apical meristem and cotyledons from damage when penetrating through the soil (Raz and Ecker, 1999). The apical hook is an exceptionally dynamic structure formed by gradual bending of the apical part of the hypocotyl soon after germination, it is maintained in a closed stage while the hypocotyl penetrates through the soil, and rapidly opens upon light exposure (Raz and Ecker, 1999; Vandenbussche et al., 2010; Žádníková et al., 2010). All phases of the hook development depend on the phase-specific coordination of differential growth and cell division (Raz and Koornneef, 2001). For the summary of the common and different factors involved in the regulation of hook asymmetrical growth see the **Table 1**.

### Apical Hook Formation

Apical hook formation is orchestrated by differential growth along the apical-basal axis of the hypocotyl, with distinct contributions of cell elongation and cell division on the opposite sides of the structure. A few, but significant, cell division events are localized at the apical concave (inner) side of the hook, while cell elongation is reduced and the arc length is shorter. In contrast, cell elongation is an active process at the convex (outer) side of the hook that permits the arc length to increase. At the basal side, the cell elongation rate is equal between the outer and inner sides and, therefore, without curvature. Thus, coordinated differential cell elongation and cell division mechanisms are essential for proper hook development (Silk and Erickson, 1978; Raz and Koornneef, 2001). Hence, bending of the apical part of the hypocotyl during hook formation is reminiscent of root and shoot tropic responses. Similar to tropic responses, an asymmetric distribution of auxin was found to be indispensable for apical hook formation. During apical hook formation, auxin has been shown to define the concave side of the apical curvature, based on polar auxin transport (Raz and Ecker, 1999). Treatment with the auxin eﬄux inhibitor NPA or the auxin influx inhibitor 1-naphtoxyacetic acid (NOA) abolishes the asymmetric auxin distribution and hook formation (Vandenbussche et al., 2010; Žádníková et al., 2010). Detailed analyses of the components of the polar auxin transport machinery based on mutant phenotype characterizations and expression studies helped to elucidate the key auxin influx and eﬄux carriers involved in the regulation of auxin transport during apical hook formation. Expression patterns, and membrane polarities of auxin transporters in particular, helped to outline the directions of auxin transport resulting in the formation of the auxin maximum at the concave side of the hook. According to the model, auxin synthesized in the cotyledons, the shoot apical meristem and the apical portion of the hypocotyl is transported basipetally toward the root. The latter basipetal transport is enhanced by the auxin influx carriers of the AUX/LAX family, which control the auxin flow from the cotyledons and shoot apical meristem, and by the eﬄux carriers PIN1 and PIN3, which act in the central cylinder. Lateral redistribution of auxin through the endodermis is promoted by PIN3 and further through the cortex and the epidermis by AUX1, PIN3 and PIN4. In the cortex, auxin transport is basipetally directed by PIN3 and PIN4 and in the epidermis by PIN3, PIN7, AUX1 and LAX3. Importantly, the accumulation of auxin at the concave side of the apical hook was proposed to result from a PIN asymmetry at the convex versus the concave side. The higher abundance of PIN3 and PIN4 at the cell membranes on the convex compared to concave side of the apical hook presumably enhances the draining of auxin from the outer cortex and epidermal layers and the formation of an auxin maximum at the concave side (Vandenbussche et al., 2010; Žádníková et al., 2010). Other important players in the control of hook formation are upstream regulators of auxin carrier activity. AUX1 exocytosis to the plasma membrane depends on the activity of the trans-Golgi network-localized ECHIDNA protein complex (Boutté et al., 2013). WAG2, a member of the AGC kinase family was found to phosphorylate PIN3 and to regulate establishment of the auxin maxima in the apical hook. Hence, it has been proposed that WAG2 might contribute to the regulation of hook formation through either control of PIN3 activity or polarity; although other PIN-independent mechanism cannot be excluded (Willige et al., 2012).

Although the polarity and expression patterns of auxin transporters provide indications for the direction of the auxin flow resulting in auxin maxima during apical hook formation, primary cues triggering this asymmetric pattern of auxin distribution and hook formation remain obscure. For tropic responses such as gravi-, or phototropism, both triggering stimuli and perception mechanisms are well defined. However, the trigger(s) of apical hook formation remain(s) unknown. Hypothetically, several candidate mechanisms controlling the onset of hook formation are plausible. To fit within a tightly enclosed seed coat, maturing embryos form a typical U-shaped structure with two cotyledons bent toward the embryonic root. Could this embryonic body shape predetermine incipient apical hook formation, after germination? Another factor that might contribute to the control of early apical hook formation is gravity. It has been shown that *Helianthus annuus* L. seedlings treated in the clinostat display a defective apical hook development, hence, supporting the role of gravisensing in hook formation (Macdonald et al., 1983). On the other hand, pea seedlings grown in a space station were able to form a partially closed apical hook, suggesting additional intrinsic mechanisms (Miyamoto et al., 2014). Thus, it seems that the formation of the apical hook might result from several mutually complementary mechanisms, and their identification and characterization are a challenge for future research.

To control hook formation, auxin locally accumulating at the concave side of the hook requires a functional transduction pathway to activate relevant downstream responses. Attenuation of auxin signaling by the expression of the auxin signaling repressor *axr3* at the concave side of the hook prevents hook formation (Vandenbussche et al., 2010). Similarly, auxin perception and signaling mutants, including *transport inhibitor response* (*tir1*; Dharmasiri et al., 2005), and *auxin resistant1* (*axr1*), *short hypocotyl2-2* (*shy2-2*), *massugu* (*msg2/iaa19*) and *non-phototropic hypocotyl4* (*nph4/msg1/arf7*), *auxin response factor19* (*arf19*; Lehman et al., 1996; Žádníková et al., 2010), respectively, do not form an apical hook, indicating an indispensable role for auxin signaling (Boerjan et al., 1995; Zhao et al., 2001). An important regulator of apical hook development is *HOOKLESS1* (*HLS1*), an ethylene-responsive gene that encodes an *N*-acetyltransferase. HLS1 was found to inhibit the AUXIN RESPONSE FACTOR2 (ARF2) repressor and, thus, to control the auxin response (Lehman et al., 1996). Apical hook formation is completely prevented in the loss-of-function mutant *hls1* (Li et al., 2004), whereas overexpression of *HLS1* causes apical hook exaggeration (Lehman et al., 1996). Among the auxin-responsive genes, several members of the *SMALL AUXIN UPREGULATED RNA* (*SAUR*) gene family are highly expressed in tissues undergoing differential cell expansion (McClure and Guilfoyle, 1989; Esmon et al., 2006). *Arabidopsis* seedlings expressing stabilized, auxin-inducible SAUR19-24 proteins are characterized by increased cell expansion, and impaired apical hook development, indicating that SAUR19–24 proteins function as positive effectors of cell expansion and therefore might contribute to the regulation of apical hook development (Spartz et al., 2012). Recent work of Spartz et al. (2014) provided new insights into the molecular mechanisms underlying SAUR19 action and demonstrated that SAUR19, through inhibition of PP2C-D subfamily type 2C protein phosphatases, activates plasma membrane H^+^ -ATPases to promote cell expansion.

### Light Triggered Apical Hook Opening

To ensure a timely start of photosynthesis, the apical hook opens shortly upon seedling exposure to light after emergence from the soil. For most dicotyledonous species, including *Arabidopsis*, light is the key trigger for hook opening (Liscum and Hangarter, 1993b). However, in some species light might have an opposite effect. Red or far-red light-induced hook exaggeration has been demonstrated to occur in a wide variety of species including tomato (*Solanum lycopersicum*), carrot (*Daucus carota*), and okra (*Abelmoschus esculentus*; Shichijo and Hashimoto, 2013). Those observations indicate that the effect of light on apical hook opening is controlled differently among species.

Light quality and intensity are two important factors that influence apical hook opening. In *Arabidopsis*, UV, blue, red and far-red light can effectively stimulate apical hook opening, while infrared light has no impact on apical hook development (Liscum and Hangarter, 1993a,b). Low-fluence red and blue light effectively trigger apical hook opening, and this effect is reversible by far-red light (Liscum and Hangarter, 1993b). Low UV-B doses are sufficient to inhibit hypocotyl growth and apical hook opening in etiolated seedlings, independently of phytochromes and cryptochromes (Ballaré et al., 1991; Kim et al., 1998; Li et al., 2013). A recent study demonstrated that a short light pulse is sufficient to trigger apical hook opening. However, this pulsed light effect results in an incompletely opened hook, which may reflect a self-protection mechanism of premature seedlings (Smet et al., 2014).

Apical hook opening is part of a plants’ photoresponse, which is achieved through multiple photoreceptors (Chen et al., 2004; Jiao et al., 2007). In plants, various photoreceptors absorb different light spectra: red and far-red light is mainly absorbed by phytochromes (phyA-phyE; Quail et al., 1995; Rockwell et al., 2006); UV-A and blue light are mainly perceived by cryptochromes (CRY1 and CRY2; Li and Yang, 2007) and phototropins (PHOT1 and PHOT2; Iino, 2006; Kimura and Kagawa, 2006); the UV RESISTANCE LOCUS 8 (UV8) is activated by UV-B light (Rizzini et al., 2011; Tilbrook et al., 2013). Currently, only PHYA, PHYB and CRY1 have been reported to act in the regulation of apical hook development (Reed et al., 1994; Fox et al., 2012; Smet et al., 2014). Phytochromes A and B were proposed to mediate apical hook opening in response to red and far-red light (Liscum and Hangarter, 1993b; Reed et al., 1994; Smet et al., 2014). Loss of CRY1 activity interferes with hook formation and the opening process is faster when compared to the wild type (Fox et al., 2012). Although several studies proposed the role of light signaling in the modulation of auxin transport (Wu et al., 2010) and auxin signaling (Stowe-Evans et al., 2001; Tepperman et al., 2001; DeBlasio et al., 2003; Nagashima et al., 2008; Wu et al., 2010; Kami et al., 2012), the mechanism of how phytochromes and cryptomes coordinate apical hook opening is largely unknown.

Besides phytochromes and cryptochromes, the function of other photoreceptors in apical hook development needs to be investigated. UV-B could possibly trigger apical hook opening (Ballaré et al., 1991). However, whether UVR8 or other yet-to-be identified UV-B receptor(s) control apical hook opening is unknown. Phototropins, receptors mediating phototropic responses, were so far not reported for their role in apical hook opening. In summary, although light-induced apical hook opening is well-established, the molecular mechanism of this process remains largely unknown.

## Conclusion

**FIGURE 1:**
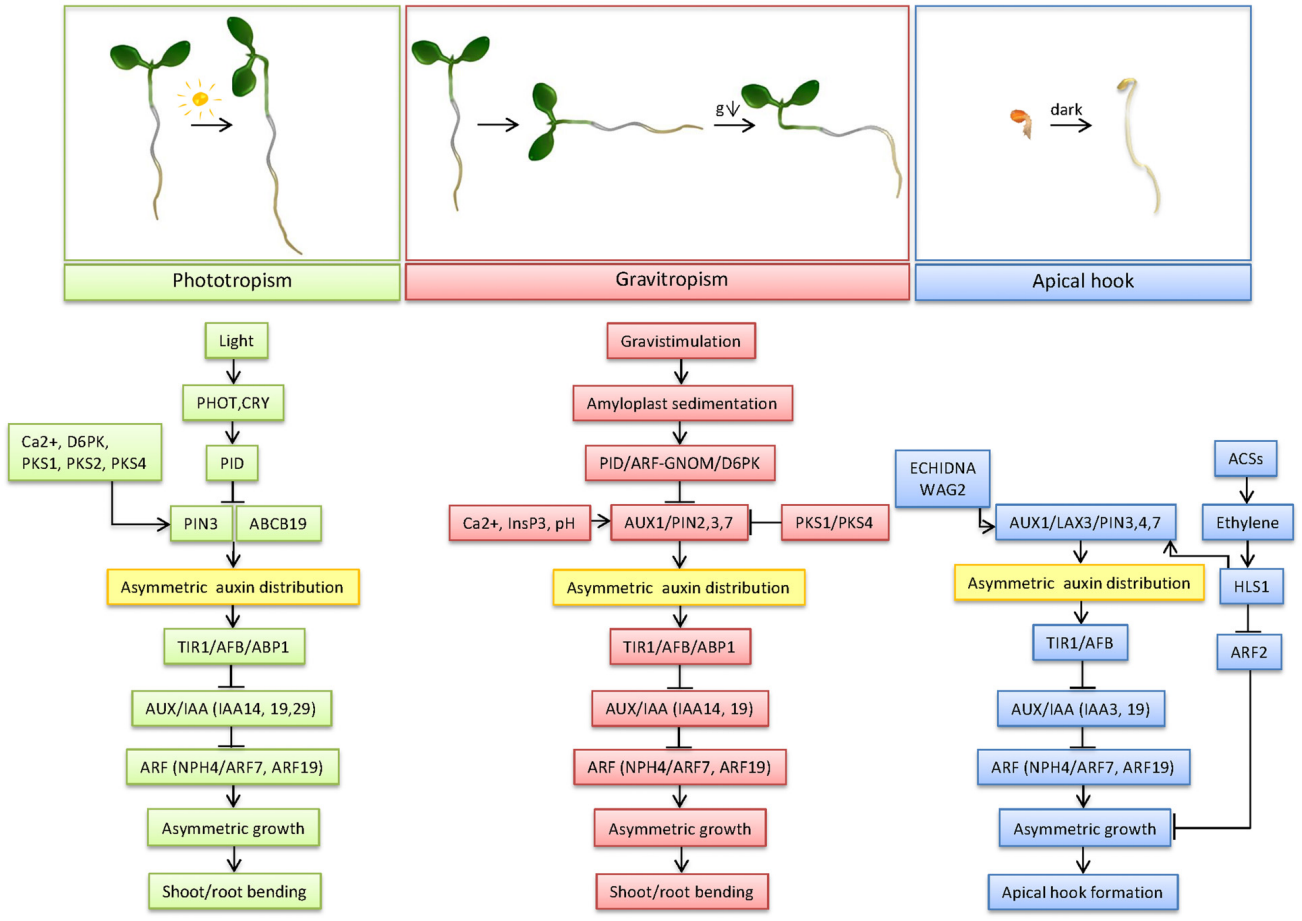
**Mechanism of differential growth in phototropism, gravitropism and apical hook development**. This model depicts the most important factors in the regulation of the three forms of differential growth, from trigger sensing to bending of the concerned organs, centered around auxin transport and signaling as key mechanisms underlying differential growth.

The ability of plant organs to bend in response to different environmental stimuli represents one of the most crucial adaptive mechanisms compensating for the lack of plant locomotion. Differential growth is the common mechanistic basis underlying all forms of tropic movements in plants. Deciphering the molecular components and mechanisms underlying differential growth in response to various environmental stimuli revealed that multiple common elements from perception to signal transduction, as well as specific elements are involved (**Table 1**; **Figure 1**). The perception sites for light and gravity differ for the shoot and the root. In addition, the response to these two stimuli rests on different mechanisms. In phototropism, light is perceived through photoreceptors, whereas in gravitropism, gravity is sensed through the sedimentation of starch-containing amyloplasts in statocytes. The triggers and perception site for apical hook formation remain, however, in the dark. To transduce the perception of both light and gravity in organ bending, common, as well as different, biochemical signals and factors are involved. Calcium ions and InsP3 appear as important common biochemical signals in both tropic responses. The increase in cytoplasmic pH in the root cap columella cells in response to gravity is another important biochemical signal, important more specifically for root gravitropism.

The difference between the perception and the response sites in both gravitropism and phototropism indicates that a signal must be transported between both sites. The polar transport of the plant hormone auxin is indeed indispensable for the differential growth underlying gravitropism, phototropism, as well as apical hook development. In all forms of differential growth, an asymmetrical auxin distribution is found in the bend of the root and the shoot. Despite the firmly established strong correlation between an asymmetric auxin distribution and differential growth, there is a longstanding enigma with respect to auxin accumulation and cell growth patterns (**Table 1**; **Figure 1**). Whereas during root gravitropism and apical hook formation auxin accumulates at the inner side of the curvature to restrict cell expansion, shoot gravi- and phototropism and presumably also root phototropism are driven by auxin maxima at the outer side of the curvature, where auxin stimulates cell growth. This apparent discrepancy in auxin accumulation patterns and consequences for local cell growth indicate that auxin might coordinate differential growth through specific mechanisms, which might depend on the developmental context. In addition, auxin is known to regulate cell growth in a concentration-dependent way. At optimal concentrations, auxin stimulates, whereas at supraoptimal concentrations it inhibits cell growth, respectively (Taiz and Zeiger, 2006). Hence, depending on the concentration of auxin, different cell growth responses might be triggered. The molecular basis of the concentration-dependent effects of auxin on cell growth are so far unknown. It might be envisioned that specific circuits within the TIR1/Aux-IAA/ARF network, which in *Arabidopsis* consists of 6 TIR1/AFBs, 29 Aux/IAAs and 23 ARFs homologues are activated in dependence on the auxin concentrations to mediate certain developmental responses. Such a scenario is strongly supported by the recent study of Calderón Villalobos et al. (2012) demonstrating that the formation of specific TIR1/AFB–Aux/IAA receptor pairs is auxin concentration dependent.

The dependence of all forms of differential growth on an asymmetrical auxin distribution indicates that auxin carriers fulfill an indispensable role. PIN3 eﬄux carriers appear to be one of the key components of the polar auxin transport machinery involved in gravitropism, phototropism and apical hook formation (Friml et al., 2002; Žádníková et al., 2010; Ding et al., 2011; Rakusová et al., 2011; Haga and Sakai, 2012; Zhang et al., 2013). In the shoot endodermal cells, light, as well as gravity, stimulate pathways targeting the PIN3 auxin eﬄux carriers and modulating their activity. Similarly, in root columella cells, the PIN3 polarity is changed in response to both light and gravity. Hence, PIN3 proteins are utmost susceptible to changes in light and gravity and promote the redirection of the auxin flow. This implies that pathways mediating qualitatively highly different external inputs might partially overlap and crosstalk to coordinate and/fine-tune responses of organs to different stimuli. Apart from PIN3, other, both different and common, auxin carriers are also involved in the three forms of differential growth. In root gravitropism, PIN2 is, together with AUX1, involved in the basipetal transport of auxin from the lateral root cap and epidermis to the root elongation zone, whereupon auxin is asymmetrically distributed by PIN3. In addition, the auxin eﬄux carriers PIN2 and PIN1 are also important for negative root phototropism (Wan et al., 2012; Zhang et al., 2014). Apart from root gravitropism, AUX1 is also important for apical hook development, where it regulates the lateral auxin distribution through the cortex and epidermis and basipetal auxin transport in the epidermis together with PIN3, PIN7 and LAX3 (Vandenbussche et al., 2010). PIN7 is also active in root gravitropism. Apical hook opening after light exposure of the shoot relies on ABCB transporters, among which importantly ABCB19 plays a prime role (Wu et al., 2010). This involvement of ABCB transporters also applies consistently to light-induced shoot phototropism (Christie et al., 2011).

Interestingly, several common components of the auxin signaling pathway, such as ARF7/NPH4 and ARF19 as well as IAA14, and IAA19, were found to control the bending of different organs regardless of the nature of the stimulus. This further strengthens the assumption that the developmental context might play a crucial role in defining the specificity of the response. The current knowledge on downstream molecular components and mechanisms which direct differential cell growth during specific tropic responses downstream of auxin is surprisingly scarce. Hence, the identification of factors and detailed understanding of networks coordinating differential growth remain a challenge for future research.

## Conflict of Interest Statement

The authors declare that the research was conducted in the absence of any commercial or financial relationships that could be construed as a potential conflict of interest.
